# Prognostic role of microRNA-145 in various human malignant neoplasms: a meta-analysis of 18 related studies

**DOI:** 10.1186/1477-7819-12-254

**Published:** 2014-08-09

**Authors:** Jie Yang, Jia-yi Zhang, Jing Chen, Chen Chen, Xiao-meng Song, Yang Xu, Jie Li

**Affiliations:** Department of Urology, First Affiliated Hospital of Nanjing Medical University, Nanjing, 210029 China; Department of General Surgery, Nanjing Hospital Affiliated to Nanjing Medical University, Nanjing, China; State Key Laboratory of Oral Diseases, West China School of Stomatology, Sichuan University, Chengdu, China; Institute of Stomatology, Department of Oral and Maxillofacial Surgery, School of Stomatology, Nanjing Medical University, Nanjing, China

**Keywords:** Malignant neoplasm, miR-145, Prognosis, Overall survival, Progression-free survival

## Abstract

**Background:**

Recent studies show that microRNA-145 (miR-145) might be an attractive tumor biomarker of considerable prognostic value. To clarify the preliminary predictive value of miR-145 for prognosis in various malignant neoplasms, we conducted a meta-analysis of 18 relevant studies.

**Methods:**

Eligible studies were identified by searching the online databases PubMed, EMBASE, and Web of Science up to March 2014. Pooled hazard ratios (HRs) with 95% confidence intervals (CIs) for patient survival and disease progress were calculated to investigate the association with miR-145 expression.

**Results:**

In total, 18 eligible studies were included in this meta-analysis. Our results showed that upregulated miR-145 significantly predicted a favorable overall survival (OS) (HR = 0.47, 95% CI 0.31 to 0.72), but failed to show a significant relation with disease prognosis. In stratified analyses, high miR-145 expression predicted favorable OS in both Whites and Asians but the intensity of the association in Whites (HR = 0.67, 95% CI 0.47 to 0.95) was not as strong as in Asians (HR = 0.35, 95% CI 0.19 to 0.64). High miR-145 expression also predicted better progression-free survival (PFS) in Asians (HR = 0.43, 95% CI 0.21 to 0.89), but not in Whites. In addition, a significantly favorable OS associated with upregulated miR-145 expression was observed in both squamous cell (SCC) (HR = 0.34, 95% CI 0.13 to 0.93) and glioblastoma (HR = 0.72, 95% CI 0.52 to 0.99).

**Conclusions:**

Our findings indicate that high miR-145 expression is better at predicting patient survival rather than disease progression for malignant tumors, especially for SCC and glioblastoma in Asians. Considering the insufficient evidence, further investigations and more studies are needed.

## Background

Emerging studies have demonstrated that deregulated expression of microRNAs (miRNAs) correlates with cancer prognosis because of the distinct expression profiles of these miRNAs in cancerous tissues compared with normal tissues [1–3]. A large number of studies have put particular emphasis on the upregulated expression of various miRNAs that are usually associated with poor prognosis in malignant neoplasms, known as ‘hazardous miRNAs’ [1–5]. However, some recent studies have transferred attention to the downregulated expression of several miRNAs that are associated with an unfavorable cancer prognosis [5–7], which are categorized as ‘protective miRNAs’ and are much less common than hazardous miRNAs. In these reported protective miRNAs, miR-145 has been studied relatively intensively and thoroughly for cancer prognosis.

**Figure 1:**
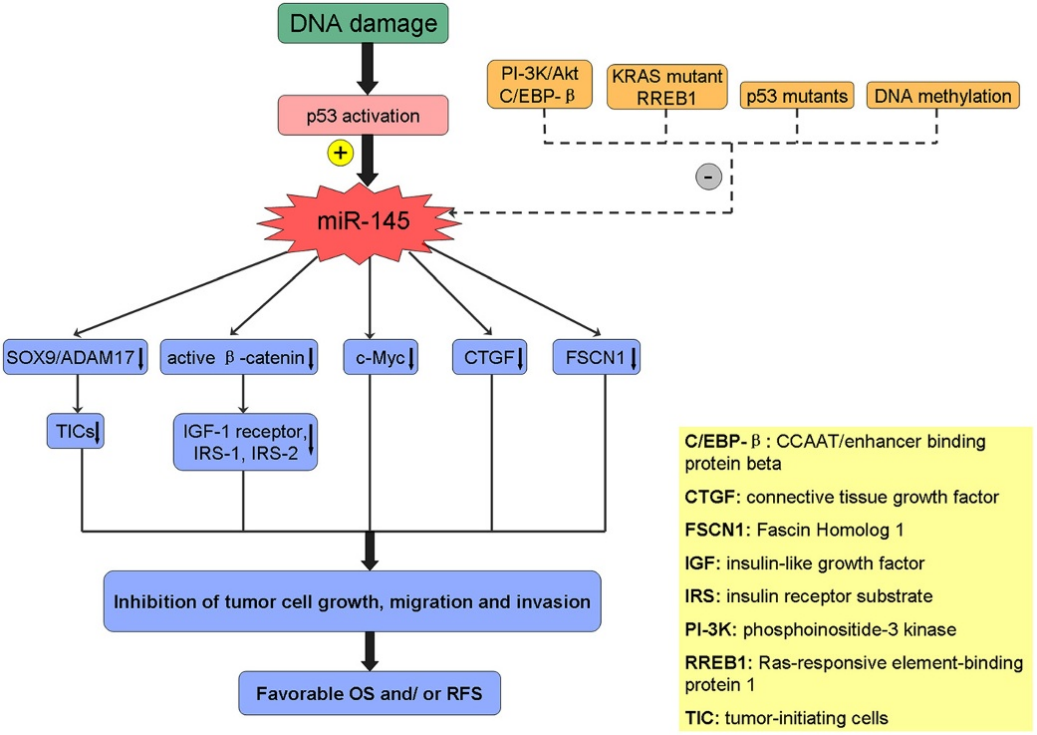
**Reported mechanisms for the anti-tumor effect and expression regulation of microRNA-145 (miR-145).**

The anti-tumor effects of miR-145 with various mechanisms have been demonstrated by abundant clinical and basic studies [8–11]. In hepatocellular carcinoma (HCC), miR-145 was found to target a number of genes along the signaling pathway of insulin-like growth factor (IGF), including IGF-1 receptor, insulin receptor substrate-1 (IRS-1), and IRS-2, all of which are directly downregulated by miR-145 [8]. Law *et al*. also confirmed that miR-145 modulates the IGF signaling pathway by reducing its downstream mediator, the active β-catenin [9]. In addition, p53 can induce miR-145 by binding directly to its promoter, after which miR-145 can silence c-Myc, demonstrating the role of miR-145 in p53-mediated c-Myc repression [10]. In head and neck squamous cell carcinoma (SCC), miR-145 also can target the SOX9/ADAM17 axis to inhibit tumor-initiating cells and IL-6-mediated paracrine effects [11] (Figure 1).

As a tumor suppressor, miR-145 has been reported to have downregulated expression in various cancer tissues, although the targets of miR-145 have not been definitively identified [8–11]. A number of studies have shown significant associations between low miR-145 expression and poor cancer prognosis, but other studies did not find any significant association, and still others showed a negative correlation [12, 13] that might cast doubt on the anti-oncogenic role of miR-145. However, in spite of these contradictory results, miR-145 is still an attractive tumor biomarker with considerable prognostic value, and deserves to be further investigated. Therefore, we conducted a meta-analysis to clarify the preliminary predictive value of miR-145 in tumor prognoses.

## Methods

### Search strategy, eligibility criteria, and data extraction

We performed this meta-analysis following the guidelines of the Meta-analysis of Observational Studies in Epidemiology group (MOOSE) [14]. Because newly published original works may get overlooked, we searched PubMed, EMBASE, and Web of Science up to March 2014 to identify relevant studies. Several combinations of the following keywords were simultaneously applied: ‘cancer’ , ‘carcinoma’ , ‘neoplasm’ , ‘tumour’ , ‘tumor’ , ‘microRNA-145’ , ‘microrna-145’ , ‘miRNA-145’ , ‘miR-145’ , ‘survival’ , ‘recurrence’ , ‘relapse’ , ‘metastasis’ , and ‘prognosis’. Studies were considered eligible for further evaluation when they met the following criteria: studies that 1) focused on patients with any malignant neoplasmI and 2) investigated the association between miR-145 expression and prognosis outcomes. In addition, the bibliographies of all eligible studies were reviewed for additional relevant publications to supplement our literature search. When studies deriving from the same series of study subjects were reported in multiple publications, only the most recent and the most complete studies were used for the meta-analysis.

**Figure 2:**
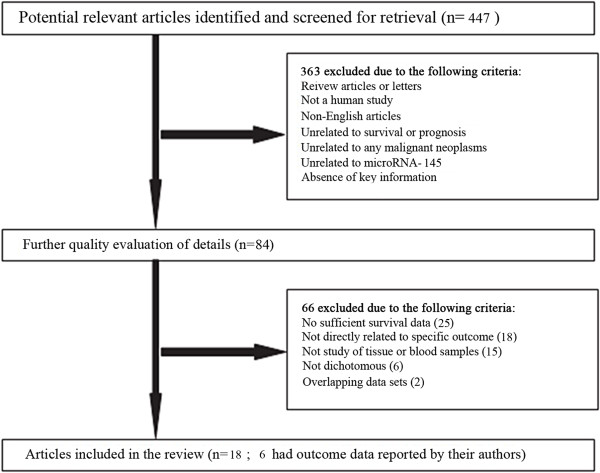
**Flow diagram of the study selection process.**

To fit the eligible criteria, selected studies had to be published in English, had to focus on human malignant tumors, and had to have performed stratified analyses on patient prognoses using the dichotomous expression levels of miR-145. Studies lacking the key data of hazard ratios (HRs) or confidence intervals (CIs), without survival curves, were not analyzed. A flow diagram of the study selection process is presented in Figure 2.

Extracted data elements included the following: 1) first author name and publication year; 2) characteristics of the studied population, including patient nationality, ethnicity, disease, pathological type, and sample category; 3) detection method and cut-off definition; 4) follow-up time; and 5) HRs of elevated miR-145 expression for overall survival (OS), recurrence-free survival (RFS), disease-free survival (DFS), metastasis-free survival (MFS), and progression-free survival (PFS), along with their 95% CIs and *P* values. If HRs and 95% CIs were not directly reported in publications, the total numbers of cases and deaths in each group were extracted to calculate HRs [15]. If only Kaplan-Meier curves were available, data were extracted from graphical survival plots to extrapolate HRs and 95% CIs, using previously described methods [16, 17]. All the aforementioned data are comprehensively detailed in Table 1 and Table 2.

**Table 1:** **Main characteristics of studies included in the meta-analysis**

First author, year of publication, and reference	Patient country of origin	Dominant ethnicity	Study design	Malignant disease	Main type of pathology	Detected sample	Survival analysis	Source of HR	Maximum follow-up, months
Lee, 2013 [5]	USA	White	R	Brain glioma	Glioblastoma	Tissue	OS	Reported	120
Saija, 2013 [6]	Finland	White	R	Brain glioma	Glioblastoma	Tissue	OS	SC	135
Campayo, 2013 [7]	Spain	White	R	NSCLC	Adeno/SCC	Tissue	RFS	SC	36
Yu, 2013 [11]	China	Asian	R	HNC	SCC	Tissue	OS	SC	60
Avgeris, 2013 [20]	Greece	White	R	Prostate cancer	Adeno	Tissue	DFS	Reported	72
Tang, 2013 [21]	China	Asian	R	Osteosarcoma	Sarcoma	Tissue	OS/DFS	Reported	152
Tanaka, 2013 [22]	Japan	Asian	R	Esophageal cancer	SCC	Serum	PFS^a^	SC	39
Speranza, 2012 [23]	Italy	White	R	Brain glioma	Glioblastoma	Tissue	OS	SC	18
Kang, 2012 [24]	Korea	Asian	R	Prostate cancer	Adeno	Tissue	RFS	Reported	55
Schee, 2012 [25]	Norway	White	R	CRC	Adeno	Tissue	MFS	SC	60
Law, 2012 [9]	China	Asian	R	HCC	Adeno	Tissue	DFS	SC	144
Ko, 2012 [12]	Canada	White	R	Esophageal cancer	SCC	Tissue	DFS	SC	32
Huang, 2012 [26]	China	Asian	R	Cervical cancer	Small cell carcinoma	Tissue	OS	SC	70
Marchini, 2011 [27]	Italy	White	R	EOC	Adeno	Tissue	OS/PFS	Reported	143
Radojicic, 2011 [28]	Greece	White	R	Breast cancer	Adeno	Tissue	OS/DFS	SC	120
Leite, 2011 [13]	Brazil	White	R	Prostate cancer	Adeno	Tissue	RFS	Reported	122
Hamano, 2011 [29]	Japan	Asian	R	Esophageal cancer	SCC	Tissue	OS	SC	97
Drebber, 2011 [30]	Germany	White	R	Rectal cancer	Adeno	Tissue	OS	SC	72

Adeno, adenocarcinoma; CRC, colorectal cancer; DFS, disease-free surviva; EOC, epithelial ovarian cancer; HCC, hepatocellular carcinoma; HNC, head and neck cancer; HR, hazard ratio; MFS, metastasis-free survival; NSCLC, non-small cell lung cancer; OS, overall survival; P, prospective; PFS, progression-free survival; R, retrospective; RCC, renal cell carcinoma; RFS, relapse-free survival; SC, survival curve; SCC, squamous cell carcinoma.

^a^PFS included any of the following: DFS, MFS or RFS.

**Table 2:** **Patient survival or disease progression associated with miR-145 expression in analyzed studies**

First author, year of publication, and reference	Assay method	Cut-off value	Cases, n		OS		PFS^a^	
			High expression	Low expression	HR (95% CI)	***P***	HR (95% CI)	***P***
Lee 2013 [5]	qRT-PCR	Mean	NM	NM	0.84 (0.74 to 0.97) (M)^b^	0.017	NM	NM
Saija 2013 [6]	Microarray	Three-fold	215	53	0.67 (0.49 to 0.92) (U)^b^	<0.01	NM	NM
Campayo 2013 [7]	qRT-PCR	Mean	56	14	NM	NM	0.30 (0.12 to 0.73) (M)^b^	0.015
Yu 2013 [11]	qRT-PCR	Two-fold	125	125	0.21 (0.14 to 0.31) (U)^b^	<0.01	NM	NM
Avgeris 2013 [20]	qRT-PCR	Mean	35	27	NM	NM	0.79 (0.31 to 2.04) (M)	0.629
Tang 2013 [21]	qRT-PCR	Median	77	89	0.28 (0.11 to 0.91) (M)	0.010	0.24 (0.09 to 0.83) (M)	0.008
Tanaka 2013 [22]	qRT-PCR	Median	32	32	NM	NM	0.89 (0.38 to 2.09) (U)^b^	0.809
Speranza 2012 [23]	qRT-PCR	Median	8	7	0.23 (0.06 to 0.83) (U)^b^	0.018	NM	NM
Kang 2012 [24]	qRT-PCR	Median	NM	NM	NM	NM	0.68 (0.22 to 2.14) (U)	0.510
Schee 2012 [25]	qRT-PCR	Median	96	97	NM	NM	1.41 (0.76 to 2.59) (U)^b^	0.290
Law 2012 [9]	qRT-PCR	1.5-fold	15	32	NM	NM	0.22 (0.09 to 0.57) (U)^b^	<0.05
Ko 2012 [12]	qRT-PCR	Two-fold	13	12	NM	NM	2.56 (1.06 to 6.18) (U)^b^	0.037
Huang 2012 [26]	qRT-PCR	Mean	22	22	0.47 (0.17 to 1.33) (M)^b^	0.072	NM	NM
Marchini 2011 [27]	qRT-PCR	Median	NM	NM	0.14 (0.03 to 0.75) (M)	0.022	2.24 (0.47 to 10.59) (M)	0.309
Radojicic 2011 [28]	qRT-PCR	Mean	38	49	1.19 (0.58 to 2.45) (U)^b^	0.824	1.04 (0.52 to 2.09) (U)^b^	0.882
Leite 2011 [13]	qRT-PCR	Mean	NM	NM	NM	NM	3.35 (1.32 to 8.50) (M)	0.011
Hamano 2011 [29]	qRT-PCR	Median	49	49	0.58 (0.33 to 0.99) (U)^b^	0.023	NM	NM
Drebber 2011 [30]	qRT-PCR	Median	35	15	0.38 (0.11 to 1.31) (U)^b^	0.244	NM	NM

CI, confidence interval; DFS, disease-free survival; HR, hazard ratio; M, multivariate analysis; MFS, metastasis-free survival; NM, not mentioned; OS, overall survival; PFS, progression-free survival; qRT-PCR, quantitative real-time PCR; RFS, relapse-free survival; U, univariate analysis.

^a^PFS included any of the following: DFS, MFS or RFS.

^b^HR and 95% CI calculated by survival curve.

### Statistical analysis

The aggregation of HRs and 95% CIs were calculated following Tierney method [17]. Forest plots were used to estimate the effect of miR-145 expression on patient survival and disease progress. Heterogeneity test for pooled HRs was verified by Cochran Q-test and Higgins I-squared statistic (I^2^). Heterogeneity was considered statistically significant at *P* < 0.1 or if the percentage of I^2^ was greater than 50%; if so, the random-effects model (DerSimonian and Laird method) was applied, otherwise, the fixed-effects model (Mantel-Haenszel test) was used [18]. In addition, we also executed stratified analyses to minimize the influence of heterogeneity by classifying analyzed studies into subgroups based on similar characteristics. Publication bias was estimated by Egger linear regression test with a funnel plot [19]. All *P* values were two-sided and a *P* < 0.05 was considered statistically significant. All statistical analyses were conducted with Stata® (v11; StataCorp LP, College Station, TX, USA).

## Results

### Summary of analyzed studies

In total, 447 studies focusing on the relationship between miR-145 and cancer were identified from an initial online literature search, and 363 studies were excluded by manual screening of titles and abstracts. The full text of the remaining 84 studies was further evaluated, and finally, 18 studies [5–7, 9, 11–13, 20–30] were considered eligible for this meta-analysis (Table 1). The selection process and excluding reasons of candidate studies are shown in detail in Figure 2.

Of the 18 included studies, 11 studies in our meta-analysis were carried out with White subjects, and 7 with Asian subjects. Quantitative reverse transcription PCR (qRT-PCR) was used to measure miR-145 expression in 17 studies, while the remaining study used microarray. The malignant neoplasms studied consisted of glioma, non-small cell lung cancer (NSCLC), head and neck cancer (HNC), prostate cancer (PCa), osteosarcoma, HCC, and colorectal, esophageal, cervical, breast, and ovarian cancers. The pathological types comprised glioblastoma, adenocarcinoma, small cell carcinoma, sarcoma, and squamous cell carcinoma (SCC). Seven of the analyzed studies reported the OS of patients, eight focused on PFS (including RFS, DFS, and MFS), and three investigated both OS and PFS. All of the analyzed studies were retrospective, and maximal follow-up time ranged from 18 to 152 months. The main characteristics of analyzed studies are systematically listed in Table 1 and Table 2.

### Overall survival associated with miR-145 expression

**Figure 3:**
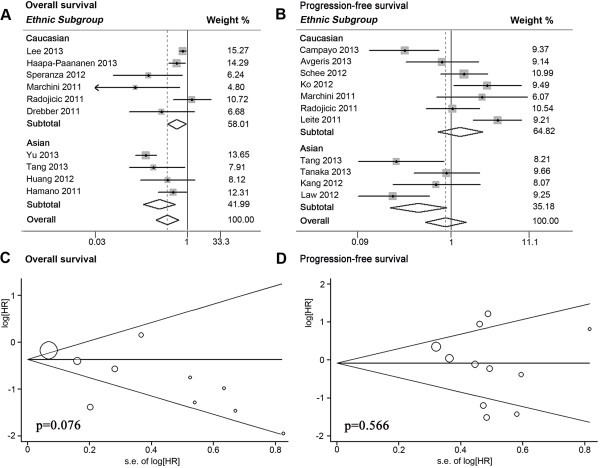
**Forest plots of merged analyses for patient survival or disease progression associated with microRNA-145 (miR-145) expression, and Begg funnel plots of publication bias test. (A)** Forest plots for overall and ethnic subtotal analyses of overall survival. Squares and horizontal lines correspond to study-specific hazard ratios (HRs) and 95% confidence intervals (CIs), respectively. The area of the squares correlates the weight, and the diamonds represent summary HRs and 95% CIs. **(B)** Forest plots for overall and ethnic subtotal analyses of progression-free survival (PFS). **(C)** Begg funnel plots of publication bias test for overall merged analysis of overall survival. Each point represents a separate study. **(D)** Begg funnel plots of publication bias test for overall merged analysis of PFS.

Ten articles analyzed OS, and seven of these showed statistical significance (Table 2). Significant heterogeneity between studies was observed (*P* < 0.001, I^2^ = 83.9%), thus a random-effects model was applied to estimate a pooled HR along with 95% CI. Our results showed that upregulated miR-145 expression was a significant predictor of favorable OS (pooled HR = 0.47, 95% CI 0.31 to 0.72) (Table 3; Figure 3A).

**Table 3:** **Patient survival or disease progression by total and stratified analyses**

Subgroup	OS			PFS^a^		
	n	HR (95% CI)	***P***value	n	HR (95% CI)	***P***value
Total	10	0.47 (0.31 to 0.72)^b^	<0.001	11	0.87 (0.51 to 1.47)^b^	0.596
Subtotal by ethnicity						
White	6	0.67 (0.47 to 0.95)^b^	0.026	7	1.27 (0.71 to 2.26)^b^	0.420
Asian	4	0.35 (0.19 to 0.64)^b^	0.001	4	0.43 (0.21 to 0.89)^b^	0.024
Subtotal by disease						
Brain glioma	3	0.72 (0.52 to 0.99)^b^	0.045	–	–	–
Prostate cancer	–	–	–	3	1.25 (0.45 to 3.48)^b^	0.671
Esophageal cancer	–	–	–	2	1.50 (0.53 to 4.22)^b^	0.443
Subtotal by main pathology						
Adeno	3	0.47 (0.14 to 1.61)^b^	0.228	7	1.01 (0.55 to 1.89)^b^	0.965
SCC	2	0.34 (0.13 to 0.93)^b^	0.035	2	1.50 (0.53 to 4.22)^b^	0.443
Glioblastoma	3	0.72 (0.52 to 0.99)^b^	0.045	–	–	–

OS, overall survival; PFS, progression-free survival including any of relapse-free survival (RFS), disease-free survival (DFS), and metastasis-free survival (MFS); N, number of studies; HR, hazard ratio; CI, confidence interval; SqCa, squamous carcinoma; Adeno, adenocarcinoma.

^a^PFS included any of the following: DFS, MFS or RFS.

^b^The HRs and 95% CIs of the analyzed studies were pooled by the random-effects model if the *P* value for heterogeneity was less than 0.10 or I^2^ was greater than 50%.

**Figure 4:**
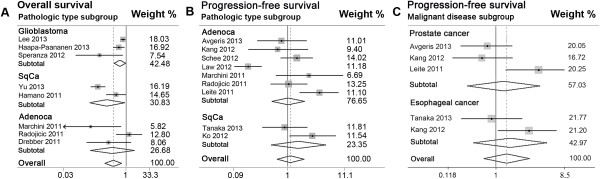
**Forest plots of merged analyses for patient survival or disease progression associated with microRNA-145 (miR-145) expression in different subgroups of pathologic types and malignant diseases. (A)** Forest plots for the merged analyses of overall survival in different pathological subgroups. Squares and horizontal lines correspond to study-specific hazard ratios (HRs) and 95% confidence intervals (CIs), respectively. The area of the squares correlates the weight, and the diamonds represent summary HRs and 95% CIs. **(B)** Forest plots for the merged analyses of progression-free survival (PFS) in different pathological type subgroups. **(C)** Forest plots for the merged analyses of PFS in different malignant disease subgroups.

Furthermore, we performed stratified analyses by classifying studies into subgroups of ethnicity and main pathologic type (Table 3; Figure 3A; Figure 4A). First, six studies in the White subgroup displayed a better OS associated with elevated miR-145 expression (pooled HR = 0.67, 95% CI 0.47 to 0.95) by a random-effects model (*P* = 0.032, I^2^ = 59.1%). The other four studies in Asians also showed that high miR-145 expression was significantly associated with a favorable OS (pooled HR = 0.35, 95% CI 0.19 to 0.64) using a random model (P = 0.025, I^2^ = 67.9%). Second, eight studies were divided into three main pathologic subgroups of adenocarcinoma, SCC, and glioblastoma. High miR-145 expression was found to be significantly associated with favorable OS in both SCC (pooled HR = 0.34, 95% CI 0.13 to 0.93) and glioblastoma (pooled HR = 0.72, 95% CI 0.52 to 0.99). No significant association was found for the adenocarcinoma subgroup (pooled HR = 0.47, 95% CI 0.14 to 1.61).

### Tumor progression associated with miR-145 expression

We analyzed tumor progression by combining disease recurrence and metastasis. Eleven studies were included in PFS analysis, and five showed statistical significance (Table 2). A random-effects model was applied to calculate the pooled HR along with 95% CI for the significant heterogeneity (P < 0.001, I^2^ = 72.9%), but failed to show any statistical significance (pooled HR = 0.87, 95% CI 0.51 to 1.47) (Table 3).

Stratified analyses displayed that high miR-145 expression was a significantly favorable prediction for tumor progression in Asian subgroup of four studies (pooled HR = 0.43, 95% CI 0.21 to 0.89) by a random-effects model (P = 0.090, I^2^ = 53.8%), but failed to show a significant association between miR-145 expression and tumor progression in White subgroup of seven studies (pooled HR = 1.27, 95% CI 0.71 to 2.26) (Table 3; Figure 3B). In addition, no significant relevance was observed in subgroups of PCa (pooled HR = 1.25, 95% CI 0.45 to 3.48), adenocarcinoma (pooled HR = 1.01, 95% CI 0.55 to 1.89), and squamous carcinoma (pooled HR = 1.50, 95% CI 0.53 to 4.22) (Table 3; Figure 4B; Figure 4C).

### Publication bias

Publication bias of total analyses for patient survival and tumor progression was evaluated by funnel plots and Egger tests. As expected, the funnel plots were symmetrical and the *P* values of the Egger test were 0.076 for OS and 0.566 for PFS, suggesting the absence of significant publication bias (Figure 3C, D).

## Discussion

Aberrant expression of miRNAs has been found in various diseases including human carcinomas, and specific miRNAs have been shown to play crucial roles in the biological behaviors of initiation, progression, migration, and invasion of tumors [31–35]. Compared with mRNAs and proteins, miRNAs are more stable and not easily degraded. They can be detected accurately by qRT-PCR in both fresh and formalin-fixed tissues [36], and can also be quantified in serum, urine, or saliva samples [37]. Therefore, miRNAs are considered promising tumor biomarkers for early diagnosis and accurate prognosis, as well as potential targets for clinical treatment [1, 3, 22, 36, 38, 39].

Recent researches have shown that downregulation of miR-145 expression is significantly associated with poor survival and prognostic outcomes of patients with cancer [5–9, 11, 21, 27, 29]. Lee *et al*. reported that low miR-145 expression in glial tumors predicted poor prognosis, and upregulated miR-145 significantly decreased the migration and invasion of glioma cells by targeting connective tissue growth factor [5]. Campayo *et al*. found that low miR-145 expression independently predicted a shorter time to relapse in patients with NSCLC [7]. In addition, downregulated miR-145 expression was found in PCa tissue compared with benign prostatic hyperplasia tissue, and this was correlated with higher Gleason score, advanced clinical stage, larger tumor diameter, and higher levels of prostate-specific antigen. The loss of the anti-oncogenic miR-145 may result in higher risk of biochemical recurrence, shorter DFS, and worse OS in patients with PCa [20]. In the present meta-analysis, we gathered the available evidence from all relevant studies to evaluate the prognostic values of miR-145 for malignant neoplasms. Our results demonstrated that high miR-145 expression was significantly correlated with favorable OS in overall analyses (*P* < 0.001), but did not exhibit an obvious association with tumor progression (*P* = 0.596) (Table 3). These inconsistent outcomes might hint at dissimilar potential mechanisms that affect patient survival or tumor progression.

Studies on miRNA expression profiles have indicated that downregulation of miR-145 is a common event in malignant disease [5, 21, 26]. However, little is known about why miR-145 is often downregulated in tumors. Recent studies show several underlying mechanisms playing key roles in regulating miR-145 expression, especially in relation to p53, the central tumor suppressor. Suh *et al*. found that downregulation of miR-145 was mediated through DNA methylation and p53 mutation pathways [40], which frequently occur in various malignant tumors. p53 can enhance post-transcriptional maturation of miR-145 in response to DNA damage, and transcriptionally inactive p53 mutants result in attenuation of miRNA biological processing activity and predict worse prognosis in patients with low miR-145 expression [41]. Loss of miR-145 expression is also observed frequently in *KRAS*-mutated pancreatic cancer, and the downregulation of miR-145 requires Ras-responsive element-binding protein (RREB1) to repress its promoter [42]. In addition, Sachdeva *et al*. suggested that a regulatory system of miR-145 involving the Akt and CCAAT/enhancer binding protein beta (C/EBP-β) may contribute to the downregulation of miR-145 in cancer cells [43] (Figure 1).

Based on the respective results of the analyzed studies, we found that genetic background, pathology, or disease type seemed to have specific effects on the association of miR-145 expression and patient prognosis. In addition, both of the overall analyses for patient survival and disease progression presented significant heterogeneity. All of these data demonstrated that the pooled results of overall analyses are crude and cannot give accurate values of miR-145 for prognosis. Stratified analyses based on ethnic affiliation, pathology, or disease categories should be carried out to minimize the impact of heterogeneity.

Our stratified analyses provide further confirmation that high miR-145 expression can predict favorable OS for patients both in White and Asian subgroups, but the association in Whites (HR = 0.67) is not as strong as in Asians (HR = 0.35). In addition, the high expression of miR-145 can predict better PFS in Asians, but not in Whites. These discrepancies might be due to different hereditary backgrounds and environmental exposure because previous studies have reported diverse expression levels and prognostic values of specific miRNAs in different ethnic groups [44–46]. Pathological types also had a considerable impact on the prognostic role of miR-145. High expression of miR-145 seemed to be predict favorable OS in patients with SCC or glioblastoma but not adenocarcinoma, but these results need to be confirmed by further research.

These results indicate that miR-145 is a promising biomarker to predict prognosis for patients with cancers. However, the conclusion is not sufficiently persuasive, and needs to be further refined for several reasons. First, no independent studies for black were included in our analysis, and this omission might hinder comprehensive investigation. Second, the number of analyzed studies was not adequate, which weakens the reliability of our results and hindered the execution of some subgroup analyses. Furthermore, all the studies included in our meta-analysis were retrospective, and no prospective studies were available, which also weakens the values of pooled results. Third, although there was no evidence of obvious publication bias in this meta-analysis, there is a possibility of language bias because only studies published in English were included. In addition, the tendency for authors and journals to publish only studies with positive results may also be a source of bias. Fourth, the expression of miR-145 was detected by tissue samples in all of the analyzed articles except one, which used serum. Different results might be obtained for detection of miR-145 in peripheral blood samples. Furthermore, as a cancer biomarker, detection of miR-145 ub serum samples is more convenient, faster and more acceptable for patients to dynamically monitor their prognosis and therapeutic effects through their lifetime. Therefore, the association between patient prognosis and serum expression of miR-145 should be further investigated. Fifth, it remains unknown whether miR-145 should be used as an independent biomarker or as part of a combination of several biomarkers for predicting tumor prognosis. Using Cox proportional regression analysis, Avgeris *et al*. demonstrated that patients with PCa who had lower miR-145 expression exhibited a significantly higher risk for disease recurrence. Furthermore, this unfavorable prognosis, associated with low miR-145 expression, was independent of patient Gleason score, clinical stage, PSA levels, and age [20]. Law *et al*. also reported that miR-145 independently coordinated the regulation of many components along the IGF pathway via its multigene targets, and was an independent prognostic predictor [9]. By contrast, Huang *et al*. claimed that the downregulation of a combination of six miRNAs including miR-145 was significantly correlated with advanced stage, lymph node metastasis, and poor prognosis in small cell carcinoma of the cervix [26]. Liu *et al*. also reported that Fascin Homolog 1 (FSCN1) could be co-regulated by miR-43 and miR-145, and suggested the combination of miR-143 and miR-145 as a potential biomarker for the prognosis of esophageal cancer [47]. The combined application of miR-145 with other miRNAs might confer more specificity for predicting patient prognosis in various malignant diseases, but this hypothesis needs to be proved by more clinical research.

## Conclusions

Our meta-analysis results indicate that high miR-145 expression is more suitable as a biomarker to predict favorable patient survival rather than to predict tumor progression, especially for SCC and glioblastoma in Asians. Given the current insufficient evidence, further investigations and more studies are needed to focus on the relationship between miR-145 expression and patient prognosis.

## Abbreviations

CI: Confidence interval
DFS: Disease-free survival
HCC: Hepatocellular carcinoma
HNC: Head and neck cancer
HR: Hazard ratio
I^2^: Higgins I-squared statistic
MFS: Metastasis-free survival
miR-145: MicroRNA-145
miRNA: MicroRNA
NSCLC: Non-small cell lung cancer
OS: Overall survival
PCa: Prostate cancer
PFS: Progression-free survival
qRT-PCR: Quantitative reverse transcription PCR
RFS: Recurrence-free survival.
